# Multi-head attention-based masked sequence model for mapping functional brain networks

**DOI:** 10.3389/fnins.2023.1183145

**Published:** 2023-05-04

**Authors:** Mengshen He, Xiangyu Hou, Enjie Ge, Zhenwei Wang, Zili Kang, Ning Qiang, Xin Zhang, Bao Ge

**Affiliations:** ^1^Key Laboratory of Modern Teaching Technology, Ministry of Education, Shaanxi Normal University, Xi’an, China; ^2^School of Physics and Information Technology, Shaanxi Normal University, Xi’an, China; ^3^Institute of Medical Research, Northwestern Polytechnical University, Xi’an, Shaanxi, China

**Keywords:** masked sequence modeling, multi-head attention, functional brain networks, feature selection, task fMRI

## Abstract

The investigation of functional brain networks (FBNs) using task-based functional magnetic resonance imaging (tfMRI) has gained significant attention in the field of neuroimaging. Despite the availability of several methods for constructing FBNs, including traditional methods like GLM and deep learning methods such as spatiotemporal self-attention mechanism (STAAE), these methods have design and training limitations. Specifically, they do not consider the intrinsic characteristics of fMRI data, such as the possibility that the same signal value at different time points could represent different brain states and meanings. Furthermore, they overlook prior knowledge, such as task designs, during training. This study aims to overcome these limitations and develop a more efficient model by drawing inspiration from techniques in the field of natural language processing (NLP). The proposed model, called the Multi-head Attention-based Masked Sequence Model (MAMSM), uses a multi-headed attention mechanism and mask training approach to learn different states corresponding to the same voxel values. Additionally, it combines cosine similarity and task design curves to construct a novel loss function. The MAMSM was applied to seven task state datasets from the Human Connectome Project (HCP) tfMRI dataset. Experimental results showed that the features acquired by the MAMSM model exhibit a Pearson correlation coefficient with the task design curves above 0.95 on average. Moreover, the model can extract more meaningful networks beyond the known task-related brain networks. The experimental results demonstrated that MAMSM has great potential in advancing the understanding of functional brain networks.

## 1. Introduction

Research into the function of the human brain has garnered significant attention and has been a popular field of study for several decades. One pivotal research direction in this field is the mapping of functional brain networks (FBNs), which has become a useful way to study the working mechanisms of the brain. By providing insight into the underlying neural mechanisms of such networks, FBNs hold the potential to unravel the working of the brain (Power et al., 2010; Park and Friston, 2013; Sporns and Betzel, 2016; Jiang et al., 2021), as well as the pathogenesis of several diseases (Canario et al., 2021). Therefore, exploring FBNs is crucial for comprehending the complex dynamics of the brain and can offer an avenue for further understanding the neural processes underlying different functions.

In traditional methods, generalized linear models (GLM) (Beckmann et al., 2003; Barch et al., 2013), independent component analysis (ICA) (McKeown, 2000; Beckmann et al., 2005; Calhoun and Adali, 2012), and sparse dictionary learning (SDL) (Lv et al., 2014; Ge et al., 2016; Lee et al., 2016; Zhang et al., 2016; Shen et al., 2017; Zhang et al., 2018) have been utilized to construct functional brain networks. Moreover, other machine learning techniques have been effectively applied to fMRI data analysis, such as support vector machines (SVM) (LaConte et al., 2005; Mourao-Miranda et al., 2006) for fMRI analysis and classification, and principal component analysis (PCA) (Thirion and Faugeras, 2003; Smith et al., 2014) for fMRI data dimensionality reduction. With the advancement of deep learning technology, numerous deep learning models have been applied to fMRI data analysis and functional brain network construction. For instance, Huang et al. (2017) proposed a deep convolutional autoencoder (DCAE) to extract hierarchical features from fMRI data; Zhao et al. (2018) proposed a spatiotemporal convolutional neural network (ST-CNN) to learn temporal and spatial information from fMRI data simultaneously; Qiang et al. (2020) proposed a spatiotemporal self-attention mechanism (STAAE) (Dong et al., 2020b) for brain functional network modeling and ADHD disease classification. Additionally, Qiang et al. (2020) proposed a residual autoencoder (RESAE) (Dong et al., 2020a) for constructing task related functional brain networks. Jiang et al. (2023) introduce a Spatio-Temporal Attention 4D Convolutional Neural Network (STA-4DCNN) model to characterize individualized spatio-temporal patterns of FBNs. Yan et al. (2022) proposed a Multi-Head Guided Attention Graph Neural Network (Multi-Head GAGNN) to simultaneously model both spatial and temporal patterns of holistic functional brain networks. Experimental results have indicated that deep learning methods are effective in fMRI data modeling and brain network construction tasks, which demonstrate the significant advantages of deep learning models.

Although the methods mentioned above have shown promising results, there are still certain limitations that need to be addressed. Firstly, the current design and parameterization of models do not fully account for the characteristics of fMRI data. For instance, the same signal value at different time points may have different meanings depending on the task or state, and thus, it is crucial to exploit this information for improving model performance. Secondly, the model training process disregards some prior knowledge, such as task design curves, which could potentially enhance the efficacy and efficiency of the model. These limitations underscore the need for more advanced techniques that can tackle these challenges and improve the accuracy and applicability of fMRI analysis.

Recent research has revealed the exceptional capabilities of Transformer models (Vaswani et al., 2017) in tasks such as text analysis and prediction. One of key mechanisms of transformer is to use multi-head attention to do the processing of sequence data. By leveraging multi-head attention mechanisms, the distinctive semantics of a single word in different language contexts can be analyzed. For instance, the term “apple” could signify either a fruit or a mobile phone brand in various language contexts. Given the similarity between fMRI time series and text sequences, multi-head attention mechanisms can be employed to extract features from fMRI data. Furthermore, the growing popularity of the masked language modeling (MLM) training method in the Bert model (Devlin et al., 2018) suggests that masking-based training techniques are remarkably effective at capturing contextual information. Since there are similarities between fMRI time series and sentences, the multi-head attention mechanism and mask training method can be extended to fMRI feature extraction.

So, this manuscript proposed a novel model called the Multi-head Attention-based Masked Sequence Model (MAMSM) which utilizes a multi-head attention mechanism to scrutinize different states of voxel signals at various locations while also implementing the Masked Sequence Model (MSM) method to analyze and process the fMRI time series. Furthermore, MAMSM employs both randomly discrete and continuous masks in the masking operation to enhance the model’s learning capacity and training effectiveness. In addition to that, this study leverages prior knowledge of the task design curves and cosine similarity to construct a new loss function, resulting in improved outcomes in model training.

In order to demonstrate the effectiveness of our proposed model, we utilized data from the Human Connectome Project (HCP) (Van Essen et al., 2013) and analyzed the seven task-state datasets of 10 individuals using both individual and group average approaches. To evaluate the performance of our model, we compared it with the SDL and STAAE methods. The experimental results indicate that the FBNs extracted by our proposed model outperformed those extracted by the other methods across various task datasets. Notably, our model also detected several brain networks that were distinct from the task-state-corresponding FBNs, and we subsequently identified some networks as similar to the known resting-state brain networks. Specifically, our experimental results demonstrate that our model is highly effective in extracting features from a small amount of data, which is particularly important in the context of brain imaging research where data acquisition is often difficult, costly, and resource-intensive. A brief version of the study has been published as a conference paper in the MICCAI 2022 (He M. et al., 2022).

## 2. Materials and methods

### 2.1. Overview

**FIGURE 1:**
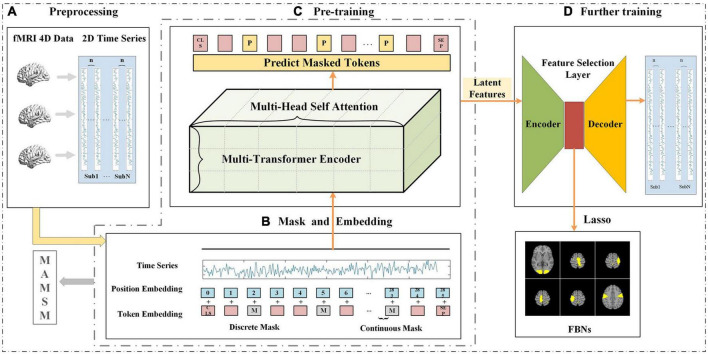
The framework of MAMSM **(A)** preprocessing, which involves mapping all subjects’ tfMRI data to 2D space; **(B)** masking and embedding operations of time-series data; **(C)** pre-training of the model, consisting of three layers of transformer encoders; **(D)** further training is performed using the feature selection layer, and the obtained features are used to map FBNs.

As shown in Figure 1, the proposed method consists of three main steps: (1) four-dimensional fMRI data is pre-processed and mapped to two-dimensional space; (2) the pre-processed two-dimensional fMRI time series is input into the MAMSM, composed of multiple headed attention mechanisms, and trained with a mask-based approach; (3) all the latent features extracted from the pre-training are input into the feature selection layer, which are trained with a loss function by leveraging the prior task designs. Finally, the features output by the encoder of the feature selection layer are regressed by lasso and mapped back to the original brain space, resulting in the visualization of FBNs.

### 2.2. Materials and pre-processing

The dataset from the Human Connectome Project Q3 was used in this work, which is publicly available on the website.^1^ We selected randomly the 10 subjects from HCP dataset. To evaluate the temporal features and spatial features obtained by the MAMSM, we chose 24 task designs from seven tasks. The corresponding hemodynamic response function (HRF) responses, which are the convolution of the task paradigm and HRF function, are utilized as temporal templates and the group-wise functional brain networks (FBNs) derived from the GLM are utilized as spatial templates (Güçlü and Van Gerven, 2017). For the sake of description, 24 distinct symbols were used to represent each of the selected task designs. For emotion task, E1 is for emotional faces, and E2 is for simple shapes. For gambling task, G1 is for punishment over baseline, and G2 is for reward over baseline. For language task, L1 is for math over story, and L2 is for story over math. For social task, S1 is for social over baseline, and S2 is for random over baseline. For relational task, R1 is for match over baseline, and R2 is for relational over baseline. For motor task, M1-M6 are for cue, left foot movement, left hand movement, right foot movement, right hand movement, and tongue movement, respectively. For working memory task, W1-W8 are for the 2-back and 0-back task events of body parts, places, faces, and tools, respectively.

The parameters of data collection used in this text is as follows: a 90 × 104 matrix, 220 mm FOV, 72 slices, TR = 0.72 s, TE = 33.1 ms, Flip angle = 52°, BW = 2,290 Hz/Px, in-plane FOV = 208 mm × 180 mm. For the tfMRI data, the pre-processing operations included skull stripping, motion correction, slice timing correction, spatial smoothing, global drift removal (high pass filtering) and registration to MNI space. Table 1 provides an overview of the pre-processed task functional magnetic resonance imaging (tfMRI) datasets used in this study. After pre-processing of the tfMRI data, the four-dimensional tfMRI data was transformed into a two-dimensional matrix by using Nilearn tools (available at https://nilearn.github.io/) and the MNI-152 mask. Data for each time point comprised 28,546 voxels.

**TABLE 1:** Summary of used datasets.

	H	W	D	Time points	Voxels	Training subjects
Motor	46	55	46	284	28,546	10
Emotion	46	55	46	176	28,546	10
Gambling	46	55	46	253	28,546	10
Language	46	55	46	316	28,546	10
Relational	46	55	46	232	28,546	10
Social	46	55	46	274	28,546	10
WM	46	55	46	405	28,546	10

### 2.3. MAMSM

#### 2.3.1. MSM

In recent years, Masked Language Modeling (MLM) and Masked Image Modeling (MIM) approaches have been widely employed in Natural Language Processing (NLP) (Devlin et al., 2018; Chung et al., 2021; Sinha et al., 2021) and Computer Vision (CV) (Zhou et al., 2021; He K. et al., 2022; Tong et al., 2022; Xie et al., 2022) due to their demonstrated efficacy in extracting contextual information through mask training. This work utilized Masked Sequence Modeling (MSM) to process fMRI sequence data. MSM is a self-supervised training method in which a portion of the tokens in the sequence are replaced with [mask] symbols and the remaining tokens and location information are used to predict the tokens replaced with [mask]. This training method allows the model to learn more about the relationships between contexts.

In the BERT model proposed by Devlin et al. (2018), the [CLS] (Classification Token) serves to create a compact representation of the entire input sequence. This condensed representation can be used for tasks such as text classification and similarity computation. Specifically, for each input fMRI time series, the proposed model is designed to generate a vector representation for each input. By adding the special [CLS] tag at the beginning of the sequence, this vector representation of the tag serves as a summary of the entire sequence, compressing and integrating the information from the entire input. As a result, the [CLS] tag provides a comprehensive representation for subsequent feature extraction and similarity calculation processes.

Before the mask processing process, the fMRI data was normalized to a range of (0, 1). After normalization, we retained three decimal places for the values, resulting in a maximum of 1,001 distinct values (from 0, 0.001, 0.002, … to 1) for the whole-brain signals. In the subsequent model training process, we treat these 1,001 different values as 1,001 classes, simplifying the model training process into a multi-classification problem. That is, if we want to predict the value of fMRI signals at a certain time point, we converted it into categories with a total of 1,001 values for classification. The prediction range of the model is also within these 1,001 classes of values. When predicting the value of a masked position, the model only needs to determine the class to which it belongs. To facilitate the prediction of token values, a multi-classification task was employed, where in a cross-entropy loss function was utilized to compute the error between the model’s predicted value and the actual value. As shown below, where **y_i_** is the true probability distribution, ${\hat{\text{y}}}_{i}$ is the predicted probability distribution, and n is the number of categories:


$$\text{C}\,E\,\left({\text{y}}_{i},{\hat{\text{y}}}_{i}\right)=-\sum_{i=1}^{n}{\text{y}}_{i}\,\text{l}\,o\,g\,\left({\hat{\text{y}}}_{i}\right)$$

In the mask processing process, for each fMRI sequence input, a certain proportion of positions on the fMRI time series will be randomly covered, with the original signal values replaced by [mask]. Here, taking the tfMRI sequence of Motor task as an example, we selected roughly 10% time points as mask locations for each input with the length of 284 time steps, as illustrated in Figure 1B. After the Mask operation was performed, the pre-training stage in the proposed model employed an unsupervised training process to predict the token values of the masked locations, as shown in Figure 1C.

In order to enhance the learning capability of the model and achieve optimal training outcomes, this study employs a combination of continuous and discrete masking techniques. When using only discrete masking, the model may be able to predict the values of the masked regions through simple methods such as averaging the values of its previous and subsequent time steps. This may lead to the model failing to learn deeper features. To avoid this issue, we designed more sophisticated methods of masking, such as continuous mask, etc., Table 2 presents the outcomes of the training with different masking modes, where 90% of the voxels in the same subject are allocated for the training set, 10% for the test set, and the same training parameters are utilized. We adopt a uniform sampling strategy for voxel selection, wherein every ten voxels, the first nine are assigned to the training set, and the last one is designated for the testing set. By comparing the minimum loss on the training set and the test set, it can be seen that the combination of the two mask operations can achieve better results.

**TABLE 2:** The results of training with different mask operations.

Mask strategies	Training loss	Predict loss
Discrete	0.043	4.73
Continuous	0.047	4.739
Discrete and continuous	0.04	4.719

#### 2.3.2. Multi transformer encoder layers

The Transformer model is a sophisticated deep neural network that is based on an attention mechanism, originally introduced by Vaswani et al. (2017) for machine translation. The model is structured according to the seq2seq paradigm and comprises two primary components: an encoder that encodes the input sequence and a decoder that generates the output sequence. Unlike traditional Recurrent Neural Network (RNN) models (Hochreiter and Schmidhuber, 1997; Schuster and Paliwal, 1997; Graves et al., 2005; Cho et al., 2014), the transformer model utilizes multi-head attention mechanism for computation. This mechanism can represent information from multiple semantic spaces, capturing different meanings of the same words in different contexts, similar to the same signal values in fMRI data may represent different states and meanings.

**FIGURE 2:**
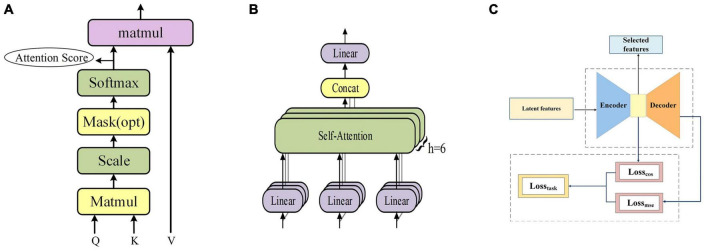
**(A)** The frame of self-attention. **(B)** The frame of multi-head attention. **(C)** The frame of feature selection layer.

Therefore, in this manuscript, each fMRI sequence is embedded and masked as the input of the transformer encoder, and then the input is linearly transformed to obtain three matrices, namely **Q** (Query), **K** (Key) and **V** (Value). Subsequently, **Q** and **K** are dot-multiplied and then normalized by dividing by $\sqrt{{\text{d}}_{k}}$ to stabilize the gradient. Subsequently, a softmax operation was used to obtain the attention score, which represents the importance of each position of the fMRI sequence, and then multiplied by *V* to obtain the output of self-attention, as shown in Figure 2A. Eventually, the output of multiple self-attentions is superimposed as the output of multi-headed attention, as shown in Figure 2B. The formulae of self-attention and multi-head attention can be expressed as follows, where **h***ead*_**i**_ denotes the ***i***-th self-attention mechanism.


$$\text{M}ultiHead\left(Q,K,V\right)=\text{C}oncat\left(\text{h}ead_{1},,\text{h}ead_{n}\right){\text{W}}^{O}$$

$$\text{h}\,e\,a\,d_{i}=\text{A}\,t\,t\,e\,n\,t\,i\,o\,n\,\left(Q,K,V\right)$$

$$\text{A}\,t\,t\,e\,n\,t\,i\,o\,n\,\left(Q,K,V\right)=\text{s}\,o\,f\,t\,m\,a\,x\,\left(\frac{{\text{QK}}^{T}}{\sqrt{{\text{d}}_{k}}}\right)\,\text{V}$$

Upon completion of the pre-training of the model, the attention score was extracted as a feature matrix, which represents the weights at various time points within an fMRI time series. After the model pre-training was completed, the attention scores were extracted as the features representing the weights of each time point in the fMRI time series. We use the sliding average operation to smooth the attention scores, and then use the average results as latent features of the pre-trained model. We set the size of the sliding average window to 10 and the step size of the sliding window is 1.

#### 2.3.3. Feature selection layer

Here we propose a novel loss function, **Loss_task_**, for the training of a feature selection layer in autoencoders, as illustrated in Figure 2C. By combining mean squared loss function (**Loss_mse_**) and cosine similarity loss function (**Loss_cos_**), this loss function is more conducive to the task of tfMRI data compared to the other methods (Dong et al., 2020b; Qiang et al., 2020), which often focus solely on reconstruction error such as MSE, disregarding the latent feature distribution and the relationship with the task curves, both of which are indispensable to characterize fMRI time series. The latent feature matrix obtained from pre-training serves as the input for the encoder, which, after training, produces the final feature matrix as its output. Through this process, the feature selection layer also facilitates the reduction of dimensionality of the latent feature matrix, thus contributing to more efficient and effective features. The **Loss_task_** function is formulated as the combination of **Loss_cos_** and **Loss_mse_** and we experimentally chose the value of k to 1 in this work, as follows:

$${\text{Loss}}_{\mathrm{mse}}=\text{M}\,S\,E=\frac{1}{\text{n}}\,\sum_{i=1}^{m}{\text{w}}_{i}\,{\left({\text{y}}_{i}-{\hat{\text{y}}}_{i}\right)}^{2}$$

$${\text{Loss}}_{\cos}={\text{l}}_{i}=1-\text{c}\,o\,s\,\left({\text{x}}_{i},{\text{y}}_{i}\right)$$

$${\text{Loss}}_{\mathrm{task}}=\text{loss}\,\left(\mathrm{mse}\right)+k*\text{l}\,o\,s\,s\,\left(\text{c}\,o\,s\right)$$

The actual value **y_i_** and the predicted value ${\hat{\text{y}}}_{i}$ are compared by calculating the cosine similarity between the n sequences of the feature**x_i_** and the n task design curves **y_i_**, and the cosine similarity calculation formula is:

$$\text{c}\,o\,s\,\left({\text{x}}_{i},{\text{y}}_{i}\right)=\frac{\sum_{i=1}^{n}\left({\text{x}}_{i}\times {\text{y}}_{i}\right)}{\sqrt{\sum_{i=1}^{n}{\left({\text{x}}_{i}\right)}^{2}}\times \sqrt{\sum_{i=1}^{n}{\left({\text{y}}_{i}\right)}^{2}}}$$

**FIGURE 3:**
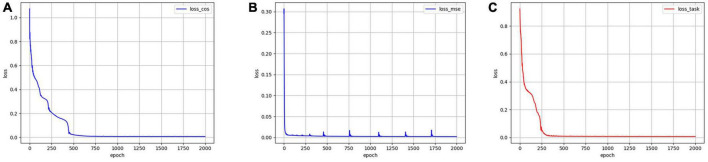
The training errors by using three different loss functions. **(A)** Error variation by using **Loss_cos_**. **(B)** Error variation by using **Loss_mse_**. **(C)** Error variation by using **Loss_task_**.

Mse-error is the MSE reconstruction error of the decoder output and the original data; Cos-error is the cosine similarity error between the n sequences of the encoder output feature and n task design curves. To demonstrate the effectiveness of the proposed new loss function, an ablation experiment was conducted using the same data and parameters. The model was trained using **Loss_cos_**, **Loss_task_**, and **Loss_mse_**, respectively. As illustrated in Figure 3, when **Loss_task_** was used, the convergence rate was faster and more stable than when only **Loss_cos_** or **Loss_mse_** was used. Quantitatively, Table 3 shows that when **Loss_task_** was employed, the final Cos-error and Mse-error were lower.

**TABLE 3:** The final training errors of three different loss functions.

	*Loss*_*cos*_	*Loss*_*mse*_	*Loss*_*task*_
Cos-error	0.0074	1.0632	**0.0067**
Mse-error	0.2955	0.0022	**0.0022**

The bold values represent the minimum values of each row.

#### 2.3.4. Mapping FBNs

To obtain the spatial distribution of the functional network, lasso regression is applied to the feature matrix and the original two-dimensional input data to get the sparse coefficient matrix, which represents the spatial distribution of the functional network The calculation formula of LASSO regression (Pedregosa et al., 2011) is as follows:

$$\underset{w}{\text{m}\,i\,n}\frac{1}{2\,\text{T}}\,{∥\text{Z}-\text{X}\,W∥}_{2}^{2}+\lambda \,{∥W∥}_{1}$$

**Z** is the original 2D input data, ***T*** represents the total number of time points, ***X*** is the feature matrix, and ***W*** is the regressed sparse coefficient matrix. The coefficient matrix ***W***, which captures the spatial distribution information of the underlying functional network, was then mapped back to the original 3D brain image space, the result was finally visualized as FBNs.

## 3. Results

The work reports its findings in terms of two primary dimensions: temporal and spatial features. To evaluate temporal features, the final feature matrix was utilized to obtain partial task-related features, which were subsequently evaluated for similarity with the task design curves. Spatial features were assessed by computing the similarity between the derived FBNs and the templates derived from the GLM. Besides task-related FBNs, we also identified additional FBNs, including those resting-state FBNs.

### 3.1. Temporal features

**FIGURE 4:**
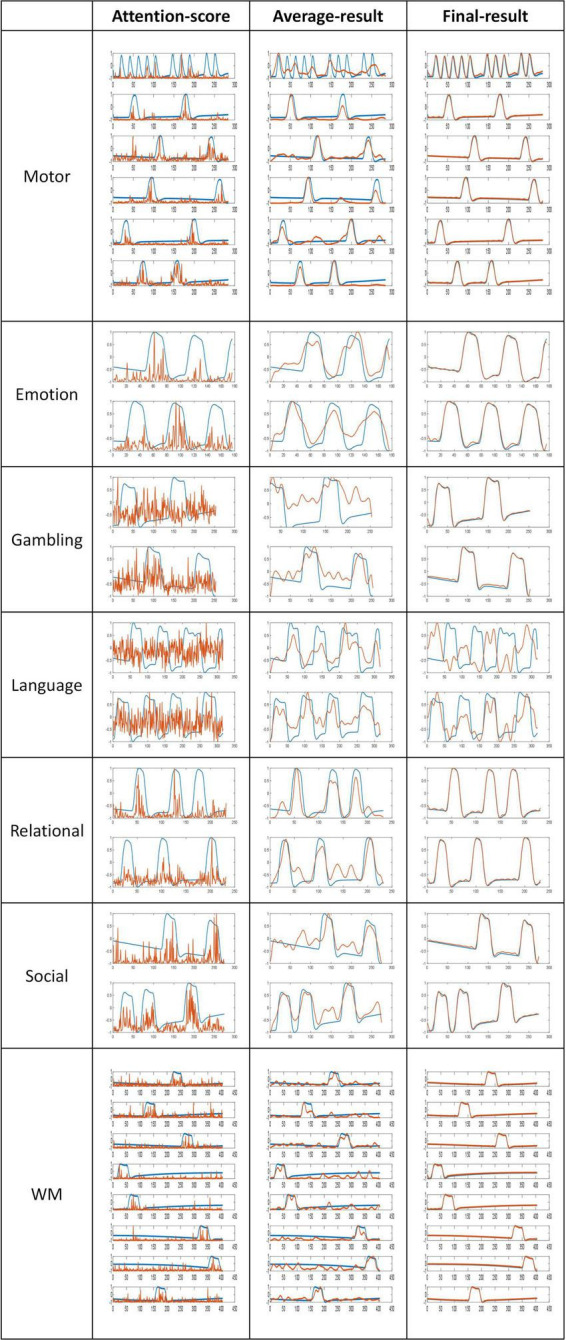
Comparison of features and task designs. The blue curves represent the task designs, the red curves depict the features.

The proposed model generated three different temporal feature matrices, namely the intermediate “attention-score” feature, which is obtained immediately after model pre-training; the “average-result” feature, calculated by computing a sliding average of the attention-score feature; and the “Final-result” feature, obtained after training the feature selection layer. The dimension of attention-score, average-result, and final-result are [6*28,546,t], [6*28,546,t], and [256,t]. In this work, “t” represents the length of the fMRI sequence’s time dimension corresponding to different tasks, while “6” denotes the number of attention heads we have set for the multi-head attention mechanism. To evaluate the significance of the three kinds of features selected in this study, a comparative analysis is conducted between these features and the task design curves. As illustrated in Figure 4, a graphical representation of the three kinds of features and the correspondingly relevant task design curves are presented. The blue curves represent the task design curves and serve as the baseline, the red curves depict the features.

Based on the results of the comparison, it is evident that the attention-score and task curves display an obvious fitting trend, with their highest peak approximately coinciding with the peak of the task design curves. Furthermore, the application of a sliding average filter results in an even higher similarity between the average-result and task design curves. These outcomes provide evidence that the latent features derived from the pre-training module are both meaningful and interpretable.

In order to quantitatively compare the similarity between the feature matrices and the task design curves, the Pearson correlation coefficient was calculated in this work, the formula for the Pearson correlation coefficient is presented below:

$$\rho \,\left(X,Y\right)=\frac{\sum_{i=1}^{n}\left({\text{X}}_{i}-\mu_{X}\right)\,\left({\text{Y}}_{i}-\mu_{Y}\right)}{\sqrt{\sum_{i=1}^{n}{\left({\text{X}}_{i}-\mu_{X}\right)}^{2}}\,\sqrt{\sum_{i=1}^{n}{\left({\text{Y}}_{i}-\mu_{Y}\right)}^{2}}}$$

where **X**,**Y** are the features and task design curves, **μ_X_**, **μ_Y_** are the mean of the them and**X_i_**, **Y_i_** are the samples of them.

The Pearson correlation coefficient values serve as an indicator of the strength of the correlation, with higher values indicating stronger correlations. As shown in Table 4, all Pearson’s correlation coefficients achieved statistical significance at the level of *P* < 0.05. These results demonstrate that the features extracted by the proposed pre-training model were significantly correlated with the design curves. Specifically, the initially extracted attention-score feature exhibited a certain degree of similarity with the task design curves. With the application of the sliding average technique, the Average-result feature approached the task design curves more. Finally, the incorporation of a feature selection layer and a new loss function as a guide led to the generation of the Final-result feature. The Pearson correlation coefficient for the task design curves was significantly improved from 0.831 to 0.975 as a result. These findings underscore the importance of the pre-training model and feature selection layer, and provide further support for the efficacy and interpretability of the proposed model in this study.

**TABLE 4:** Pearson correlation coefficient between the features and the task designs.

	E1	E2	G1	G2	W1	W2	W3	W4	W5	W6	W7	W8	/
Attention-score	0.331	0.343	0.321	0.333	0.287	0.268	0.270	0.267	0.276	0.262	0.275	0.276	/
Average-result	0.851	0.894	0.792	0.792	0.813	0.799	0.870	0.804	0.845	0.845	0.789	0.856	/
Final-result	0.999	0.998	0.998	0.998	0.999	0.999	0.999	0.994	0.999	0.999	0.998	0.999	/
	**L1**	**L2**	**S1**	**S2**	**R1**	**R2**	**M1**	**M2**	**M3**	**M4**	**M5**	**M6**	**Ave**
Attention-score	0.262	0.250	0.319	0.583	0.419	0.337	0.336	0.423	0.430	0.408	0.427	0.567	0.345
Average-result	0.737	0.785	0.796	0.916	0.880	0.889	0.451	0.903	0.913	0.880	0.873	0.961	0.831
Final-result	0.727	0.737	0.998	0.997	0.999	0.999	0.973	0.997	0.998	0.997	0.997	0.997	0.975

### 3.2. Spatial features

#### 3.2.1. Task FBNs

**FIGURE 5:**
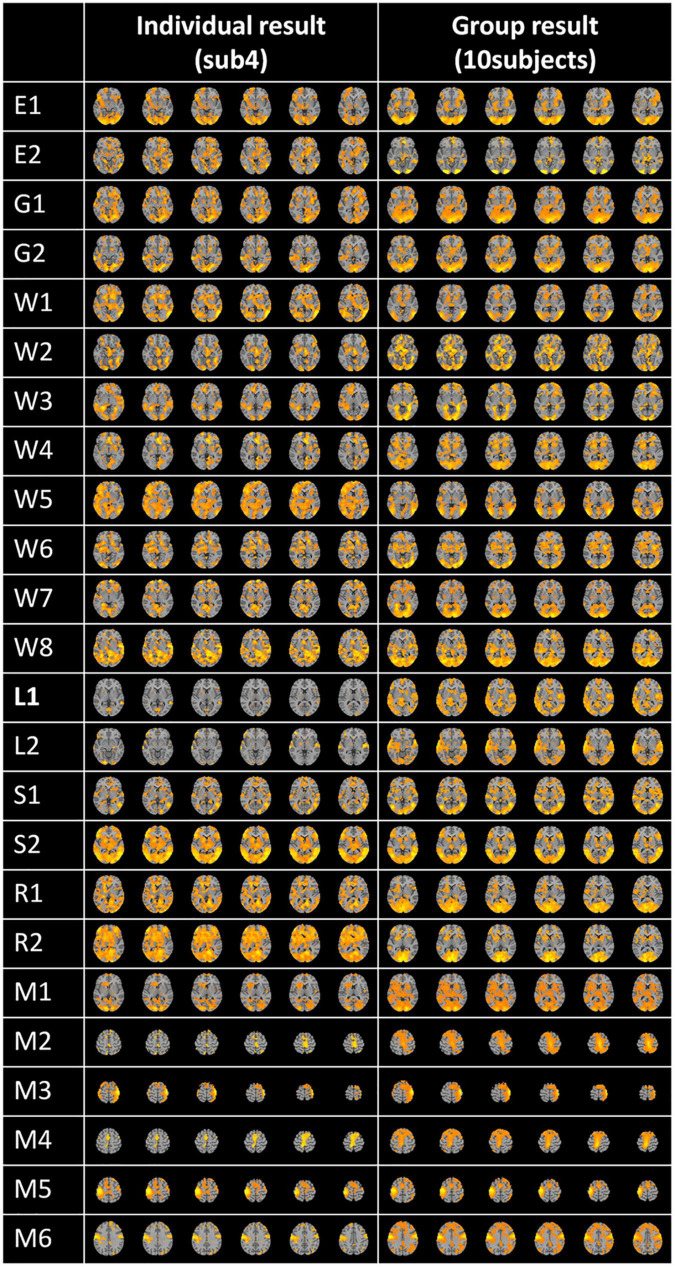
Individual and group averaged FBNs.

Following the feature selection process, the feature matrix was remapped to the original 3D brain space for the visualization of FBNs using lasso regression, as shown in Figure 5. This figure displays a randomly selected individual FBN for 24 tasks and group-averaged FBNs from 10 subjects. As demonstrated in Figure 5, each task-related FBN can be accurately identified, and the FBNs becomes even more pronounced after group averaging.

#### 3.2.2. Other FBNs

**FIGURE 6:**
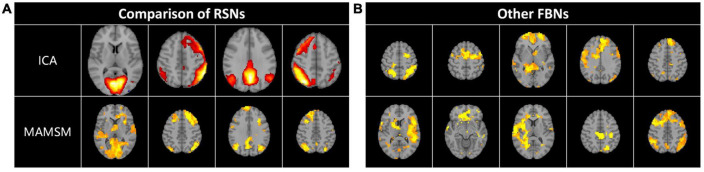
**(A)** Some resting-state FBNs. **(B)** Other FBNs.

Multi-head Attention-based Masked Sequence Model can not only acquire the known activated networks, but also enable the identification of other brain networks with specific patterns. In this work, we also selected and displayed a part of them. After comparison and analysis, we found some resting-state networks, which were compared and displayed with the corresponding resting-state brain network templates obtained by the ICA method, as shown in Figure 6A. In addition, this manuscript also displays other brain networks with certain patterns, as shown in Figure 6B.

### 3.3. Comparative experiments

To further evaluate the effectiveness of the proposed MAMSM, it is compared with SDL (Lv et al., 2015)and STAAE (Dong et al., 2020b). SDL is the traditional way to build FBNs. STAAE has been proposed as a deep learning method recently. All three methods are applied to the same dataset and their temporal and spatial characteristics are compared in this section.

#### 3.3.1. Comparison of temporal features

**FIGURE 7:**
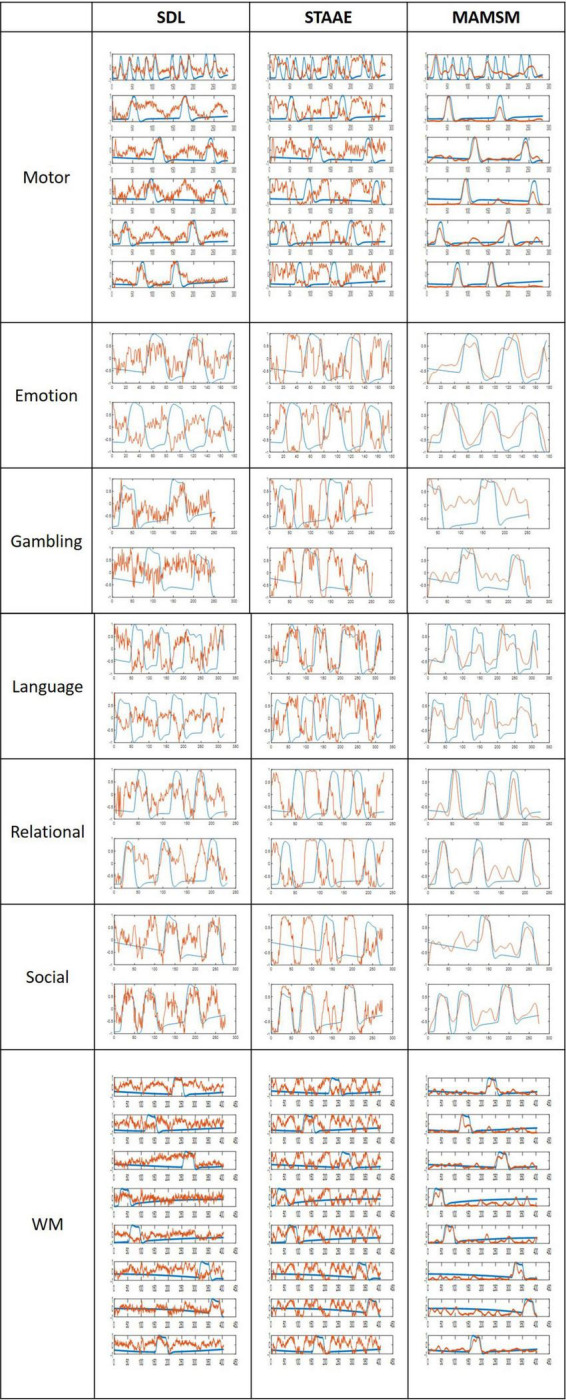
Comparison of features and task designs obtained by different methods. The blue curves represent the task designs, the red curves depict the features.

In this study, three different methods were employed for comparison purposes. In order to ensure fairness in our comparison analysis, we adopted the “average-result” features instead of the “final result” features for comparison with the features obtained from SDL and STAAE, as our proposed model leveraged prior knowledge (task designs) to train the model in the feature selection layer. Figure 7 displays the task design curves, with the blue curves representing specific task design curves used as comparison benchmarks and the red curves representing the task-related features. Our qualitative and quantitative comparison analysis aimed to assess the degree of correlation between these two curves. For quantitative comparison, the Pearson correlation coefficient was employed to assess the similarity between the extracted features and the task curves, as presented in Table 5. It should be noted that Figure 7 shows the results of an individual. Table 5 is the average result of ten individuals.

**TABLE 5:** Pearson correlation coefficient obtained by SDL, STAAE, and MAMSM.

	E1	E2	G1	G2	W1	W2	W3	W4	W5	W6	W7	W8	
SDL	0.631	0.624	0.483	0.515	0.390	0.356	0.395	0.443	0.419	0.369	0.453	0.379	/
STAAE	0.322	0.246	0.351	0.385	0.195	0.128	0.259	0.272	0.088	0.069	0.197	0.155	/
MAMSM	0.830	0.867	0.848	0.821	0.864	0.870	0.869	0.803	0.849	0.819	0.799	0.869	/
	**L1**	**L2**	**S1**	**S2**	**R1**	**R2**	**M1**	**M2**	**M3**	**M4**	**M5**	**M6**	**Ave**
SDL	0.603	0.622	0.523	0.673	0.514	0.564	0.658	0.603	0.586	0.493	0.603	0.738	0.527
STAAE	0.606	0.619	0.302	0.651	0.440	0.422	0.429	0.218	0.203	0.189	0.226	0.383	0.306
MAMSM	0.760	0.777	0.812	0.835	0.863	0.868	0.500	0.836	0.862	0.838	0.850	0.875	0.824

As shown in Figure 7, the correlation between the features generated by MAMSM and the task design curves was found to be significantly higher compared to that between the features generated by SDL/STAAE and the task design curves. Quantitatively, the results presented in Table 5 demonstrate that the proposed MAMSM achieved a higher averaged Pearson correlation coefficient (0.824) compared to that from SDL (0.527) or STAAE (0.306). Overall, the results of our experiment demonstrate the effectiveness of MAMSM for constructing FBNs based on tfMRI.

In terms of individual-level performance, our results indicate that the deep learning method STAAE performed slightly worse than SDL and MAMSM. It is worth noting that according to the description of the STAAE (Dong et al., 2020b), the method can achieve better results when applied to larger datasets. However, the inherent requirement of deep learning methods for large volumes of data may limit their advantage over traditional methods in cases where data availability is limited. Our proposed method, on the other hand, demonstrates good performance on individual data, suggesting that it can effectively learn temporal features from small datasets.

#### 3.3.2. Comparison of spatial features

**FIGURE 8:**
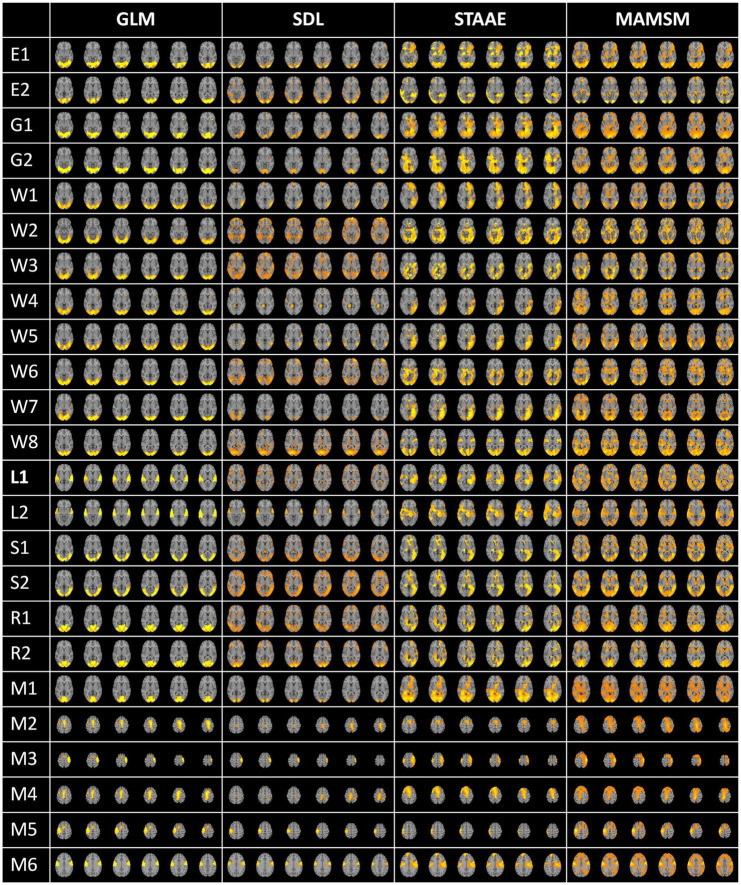
Comparison of FBNs obtained from SDL, STAAE, and MAMSM.

In order to qualitatively compare the spatial features from the three methods, this work applies SDL, STAAE, and MAMSM to the same dataset and obtains the group averaged results, as shown in Figure 8. The GLM templates were derived by summarizing a large amount of individual data and were subsequently employed for the purpose of comparing the performance of FBNs generated through various methods. Our results demonstrate that the activation maps obtained through MAMSM exhibit greater resemblance to the GLM templates.

Quantitatively, we also used the spatial overlap rate as an indicator to compare the FBNs from the three methods and the GLM template. The spatial overlap rate can be used to compare the similarity between two different networks, which is defined as follows:

O⁢R⁢(N1,N2)=∑i=1n|Ni1∩Ni2|∑i=1n|Ni1∪Ni2|

*N*1, *N*2 are the two brain networks to be compared, *n* is the number of voxel points of the brain network. The spatial overlap rate of the FBNs obtained from each method and GLM templates are shown in Table 6. We can see that the average OR value (0.295) of the brain network obtained by MAMSM is larger than that of STAAE (0.231) and SDL (0.202), which proves that the MAMSM proposed in this manuscript is superior to STAAE and SDL.

**TABLE 6:** The spatial overlap rate obtained by SDL, STAAE, and MAMSM.

	E1	E2	G1	G2	W1	W2	W3	W4	W5	W6	W7	W8	
SDL	0.150	0.102	0.231	0.200	0.231	0.236	0.266	0.236	0.203	0.225	0.226	0.262	/
STAAE	0.188	**0.234**	0.265	0.210	0.186	0.247	0.209	0.172	0.200	0.263	0.241	0.200	/
MAMSM	**0.221**	0.171	**0.321**	**0.320**	**0.274**	**0.262**	**0.302**	**0.288**	**0.213**	**0.307**	**0.256**	**0.293**	/
	**L1**	**L2**	**S1**	**S2**	**R1**	**R2**	**M1**	**M2**	**M3**	**M4**	**M5**	**M6**	**Ave**
SDL	0.209	0.177	0.272	0.273	0.244	0.201	0.133	0.146	0.122	0.143	0.146	0.206	0.202
STAAE	0.210	0.265	0.161	0.199	0.205	0.206	0.302	0.293	0.257	0.272	0.273	0.293	0.231
MAMSM	**0.305**	**0.272**	**0.352**	**0.374**	**0.374**	**0.258**	**0.343**	**0.345**	**0.299**	**0.297**	**0.314**	**0.322**	**0.295**

The bold values represent the maximum values of each column.

## 4. Discussion and conclusion

In this study, the multi-head attention mechanism and mask training method were applied to the analysis of tfMRI data, and a new loss function was constructed by task design curves for the mapping of functional brain networks. The multi-head attention mechanism helps the model better understand the situation where the same signal value in tfMRI signals may represent different states. Meanwhile, a mask training method was adopted to learn the relationship between the contexts of input sequences, and by combining a continuous mask and a discrete mask, deeper-level features were learned. The experimental results demonstrated that these techniques can improve the model’s performance. By analyzing the comparison results of the intermediate features (attention-score, average-result) outputted from the model and the task design curves, it can be seen that the proposed model can better understand the tfMRI signals and the derived features are interpretable. The attention-score extracted after the model was trained represented the weight scores of different locations in each tfMRI sequence. The region with the highest score in the attention-score bears close resemblance to the area with the most significant alteration in the task design curves. The average-result obtained by simply sliding the attention-score achieved higher similarity with the task design curves than the results obtained by other methods.

We also leveraged prior knowledge (Task designs) to guide the model to learn the more efficient features, the task designs were introduced to build a new loss function which optimizes the model by cosine similarity error and MSE error. By analyzing the results, we found that this new loss function can improve the performance of the model. Other methods usually ignored the prior knowledge in their model, and experimental results show that MAMSM achieves better results than other methods when using the new loss function.

The experimental results show that the proposed method can achieve better generalization performance on smaller sample size, compared to other deep learning methods which require large amounts of data to achieve better results, such as STAAE (Dong et al., 2020b), ResAE (Dong et al., 2020a), Dvae (Qiang et al., 2020) and so on. Due to the characteristics of medical image data, such as high confidentiality and small sample size, the method proposed in this manuscript can have better development prospects in the future.

It is important to note that this study has certain limitations. Firstly, the relatively small size of the dataset employed may introduce noise when aggregating across groups, potentially impacting the outcomes of the brain network analyses. Furthermore, the present methodology places greater emphasis on temporal features of tfMRI data, and future investigations may benefit from incorporating a combination of convolutional neural network (CNN) models (Ronneberger et al., 2015; Liu et al., 2022) and visual transformer (VIT) models (Dosovitskiy et al., 2020; Liu et al., 2021) to extract spatial features, which may achieve better results. Additionally, the precise functional significance of some brain networks identified in the results is not fully understood at present, and hence, further research is warranted to explore the functional areas and meanings attributed to these networks.

## Data availability statement

Publicly available datasets were analyzed in this study. This data can be found here: https://humanconnectome.org/study/hcp-young-adult/document/q3-data-release.

## Ethics statement

The studies involving human participants were reviewed and approved by the dataset is public and has been approved by its Ethics Committee. The patients/participants provided their written informed consent to participate in this study.

## Author contributions

MH: methodology, formal analysis, software, visualization, writing—original draft, and writing—review and editing. XH: visualization and writing—original draft. EG, NQ, and XZ: writing—review and editing. ZW: software and visualization. ZK: validation and visualization. BG: conceptualization, methodology, writing—review and editing, funding acquisition, resources, and supervision. All authors contributed to the article and approved the submitted version.

## Footnotes

1
https://db.humanconnectome.org
